# Long-term Trends in Prevalence of Neural Tube Defects in Japan

**DOI:** 10.2188/jea.JE20180126

**Published:** 2019-04-05

**Authors:** Takashi Yorifuji

**Affiliations:** Department of Human Ecology, Graduate School of Environmental and Life Science, Okayama University, Okayama, Japan

**Keywords:** dietary supplements, folic acid, fortification of the food supply, neural tube defects, long-term trend, spina bifida

Neural tube defects (NTDs), major congenital anomalies of the central nervous system, result from failure of the neural tube to close between the 3^rd^ and 4^th^ week of gestation and include spina bifida, anencephaly, and encephalocele.^1^ Because previous epidemiological studies demonstrated that low folic acid intake conclusively increases the risk of NTDs, intake of folic acid is recommended worldwide for women during their reproductive years.^2^

Natural folate contained in foods has rather low and variable bioavailability compared with pure folic acid, and supplementation of folic acid is recommended to increase plasma folate levels.^2^ Accordingly, the Centers for Disease Control in the United States recommended intake of folic acid supplements in 1992 to all potentially pregnant women because the critical period for NTDs occurs before pregnancy is recognized by many women. Following this global trend, the Japanese Government in 2000 recommended intake of folic acid supplements during the period from 1 month before pregnancy to 3 months of pregnancy for potentially pregnant women.^3^ The guideline recommends intake of 400 µg of folic acid supplement per day, in addition to dietary intake of folic acids. Despite the unequivocal benefits of folic acid supplementation, use of the supplement, in particular during the recommended period, has not increased substantially. For example, a nationwide survey in Japan demonstrated that about 85% of pregnant women took folic acid supplements during pregnancy, although only 37% consumed them prior to pregnancy and more than half of them consumed the supplements even after 4 months of pregnancy.^3^ The pregnant women who did not comply with the guideline tended to be young, multigravida, lacked knowledge of the benefit of the intake, and had never used supplements (other than folic acid) before pregnancy.^3^ The primary limitation of supplementation is that not all women will comply and this is most likely to be problematic among low-income groups who could benefit the most.^2^ Therefore, fortification of the food supply with folic acid (eg, in flour or rice) has been mandatory in more than 80 countries to increase the population’s intake of folic acid, and this intervention was successful in reducing the prevalence of NTDs.^2^^,^^4^

Recent epidemiological studies also suggest that low intake of folic acid is associated with increased risk of other congenital anomalies, such as congenital heart diseases and orofacial clefts. In this issue, therefore, Ito et al examined the associations between serum folate concentrations measured at a mean of 10.8 weeks of gestation and various congenital anomalies, including NTDs, during the period from 2003 to 2012 in a large birth-cohort in Hokkaido, Japan.^5^ However, it seemed that they failed to evaluate the associations and provide meaningful information because of some bias and lack of power. I agree with their opinion that case-control or case-cohort study design is needed to evaluate the hypothesis, but I wonder why they did not evaluate the associations between intake of folic acid supplements, hopefully during the recommended period, and the outcomes, which can reduce non-differential exposure misclassification. Many previous epidemiological studies used intake of supplements as an exposure indicator, and it is uncertain whether “serum” folate intake (not red blood cell concentrations that can represent long-term levels) at 10.8 weeks of gestation can represent folate levels during the critical period of exposure.

Moreover, they discussed that only 0.7% of the participants were at the deficiency level for folic acid (less than 6.8 nmol/L in serum levels) according to the WHO criteria,^5^ but the guideline was for the population at all age groups to prevent macrocytic anemia.^6^ The required level for women of reproductive age is much higher according to the guideline. Although no serum folate threshold is recommended, the red blood cell threshold for women of reproductive age is about four times higher compared with that for the population at all age groups to define folate insufficiency.^6^ Considering that median serum folate level of the study participants was 16.6 (interquartile range, 13.4–21.5) nmol/L,^5^ most of the participants in this cohort would be classified as having an insufficient level of folic acid to prevent NTDs.

**Figure 1.**
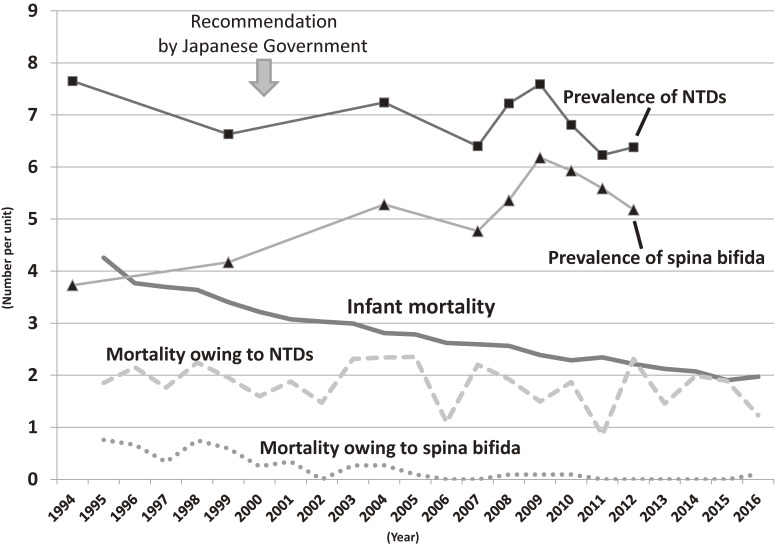
Trends in prevalence and mortality under 1 year of age of neutral tube defects (NTDs), as well as infant mortality in Japan from 1994 to 2016. The units are one per 1,000 births for infant mortality, one per 100,000 births for mortality owing to NTDs and spina bifida, and one per 10,000 births for prevalence. The International Classification of Diseases-10 codes are Q00, Q01, and Q05 for NTDs and Q05 for spina bifida. Mortality information was obtained from Vital Statistics from Ministry of Health, Labour, and Welfare in Japan. Prevalence for NTDs (■) and spina bifida (▲) information was obtained from the Annual Report 2009–2014 published by the International Clearinghouse for Birth Defects Surveillance and Research and prevalence before 2006 was average of the prevalence during the 5 years (eg, 5-year average of the prevalence from 2002 to 2006 is shown in 2004).

When we look at long-term trends of NTDs in Japan, prevalence of NTDs did not decline, despite the recommendation of supplement intake by the Japanese Government in 2000. As shown in Figure 1, mortality owing to NTDs, in particular spina bifida, declined in parallel with the decreasing trend of infant mortality, while prevalence of NTDs did not. This situation is consistent with long-term trends in the prevalence of NTDs in Europe, where mandatory fortification programs do not yet exist.^7^ Despite longstanding recommendations aimed at promoting periconceptional folic acid supplementation and existence of “voluntary” folic acid fortification, the prevalence of NTDs, including fetal deaths (around 9 per 10,000 births), did not decline over those 20 years.^7^ Some may argue that the prevalence in Japan is already low compared with that in Europe, so supplementation of folic acid is not effective. But the prevalences shown in Figure 1 do not include numbers of NTDs from fetal deaths, and a previous case-control study in Japan also demonstrated that lack of intake of folic acid supplements increased the risk of NTDs^4^; thus, folic acid supplementation would be effective even for women of reproductive age in Japan. Considering the inadequate supplement intake during the periconceptional period in Japan^3^ and the fact that the level for folic acid of most of the pregnant women observed in the Hokkaido study would be classified as insufficient, recommendation of intake of folic acid supplements would not be adequate as a public health strategy. Rather, the mandatory folate fortification program would potentially be the most effective strategy because it can reach almost the entire population and does not depend on constant education and motivation.^2^^–^^4^ Thus, reductions in prevalence of NTDs would be possible even in Japan.

What should we do as epidemiologists? It is necessary to conduct further studies to accumulate more evidence on the associations of folic acid intake and NTDs and other congenital anomalies, as Ito et al conducted. Epidemiology not only elucidates the etiology of the disease, but can also evaluate the beneficial effect of political intervention on human health. Therefore, we should return to review the trends of NTDs after several interventions. At this stage, however, when we look at the trend of NTDs in Figure 1, we would ultimately disagree with the beneficial effect of the recommendation in 2000.
